# Application of MALDI-TOF MS for enumerating bacterial constituents of defined consortia

**DOI:** 10.1007/s00253-023-12558-5

**Published:** 2023-05-06

**Authors:** Michael P. Coryell, Rosa L. Sava, Jessica L. Hastie, Paul E. Carlson

**Affiliations:** Division of Bacterial, Parasitic, and Allergenic Products; Office of Vaccines Research and Review, Center for Biologics Evaluation and Research, US Food and Drug Administration, Silver Spring, Maryland, USA

**Keywords:** Microbial Enumeration, Biologics, Live Biotherapeutic Products, Advanced manufacturing techniques

## Abstract

**Abstract:**

Characterization of live biotherapeutic product (LBP) batches typically includes a measurement of viability, such as colony forming units (CFU). However, strain-specific CFU enumeration assays can be complicated by the presence of multiple organisms in a single product with similar growth requirements. To overcome specific challenges associated with obtaining strain-specific CFU values from multi-strain mixtures, we developed a method combining mass spectrometry-based colony identification with a traditional CFU assay. This method was assessed using defined consortia made from up to eight bacterial strains. Among four replicate batches of an eight-strain mixture, observed values differed from expected values by less than 0.4 log_10_ CFU among all strains measured (range of differences, -0.318 to + 0.267). The average difference between observed and expected values was + 0.0308 log_10_ CFU, with 95% limits of agreement from -0.347 to 0.408 (Bland–Altman analysis). To estimate precision, a single batch of eight-strain mixture was assayed in triplicate by three different users, for a total of nine measurements. Pooled standard deviation values ranged from 0.067 to 0.195 log_10_ CFU for the eight strains measured, and user averages did not differ significantly. Leveraging emerging mass-spectrometry-based colony identification tools, a novel method for simultaneous enumeration and identification of viable bacteria from mixed-strain consortia was developed and tested. This study demonstrates the potential for this approach to generate accurate and consistent measurements of up to eight bacterial strains simultaneously and may provide a flexible platform for future refinements and modifications.

**Key points:**

*• Enumeration of live biotherapeutics is essential for product quality and safety.*

*• Conventional CFU counting may not differentiate between strains in microbial products.*

*• This approach was developed for direct enumeration of mixed bacterial strains simultaneously.*

**Supplementary Information:**

The online version contains supplementary material available at 10.1007/s00253-023-12558-5.

## Introduction

Live biotherapeutic products (LBPs) are products containing one or more live microorganism(s) as active substances that are intended to be used for the treatment, prevention, or cure of medical conditions (US Food and Drug Administration 2016, Dreher-Lesnick et al. 2017). In the United States, LBPs are categorized as biological products and, therefore, require development of meaningful tests for identity, purity, and potency of specific drug substances and drug products in their final dosage form. It is expected that the therapeutic effects of LBPs will rely on activity of living organisms delivered in the final dosage. As such, assays designed to measure the total viable organisms in the final product formulation (e.g., colony forming units (CFU) per dose) are often included as a measurement of product potency (Dreher-Lesnick et al. 2017, Pot et al. 2021). However, this relatively straight-forward measurement can be complicated by the presence of multiple bacterial strains in an LBP, especially if they share similar cultivation requirements and/or phenotypic traits.

In situations where selective culture is not practical or appropriate, obtaining CFU counts for multiple bacterial strains from a single mixture grown on the same medium requires identification of individual colonies in sufficient numbers to accurately determine viable amounts of each bacterial strain. Identifying hundreds of individual colonies using conventional methods like metabolic testing, colony PCR, or 16S rRNA gene sequencing can be complex, time consuming, and prohibitively expensive (Cook et al. 2003; Lao et al. 2022). However, within the field of laboratory diagnostic testing, several commercially available platforms now provide rapid and accurate microbial identification from direct analysis of colony material using matrix-assisted laser desorption ionization time-of-flight mass spectrometry (MALDI-TOF MS) technology. MALDI-TOF MS methods have been developed and implemented for identification of various bacterial, fungal, and archaeal targets isolated from numerous sources including clinical specimens, food and dairy products, and environmental samples (Singhal et al. 2015; Gong et al. 2021; Guindo et al. 2022). These methods benefit from simplified sample processing, rapid time-to-results, low cost of reagents, and amenability to use with automation tools, when compared with conventional identification methods (Chudejova et al. 2017; Theparee et al. 2018).

In this study, we assessed the utility of a newly developed method combining traditional colony counting with rapid colony identification using MALDI-TOF MS as a method for direct enumeration and identification of the viable organisms in defined mixtures of bacterial cultures. Experimental CFU measurements were compared with expected CFU values derived from pure culture enumerations, and precision was evaluated by repeated measurements of a single batch of mixture by multiple users. This study demonstrates the feasibility of this flexible approach to measuring concentrations of different viable bacteria present in mixed samples without selective or differential methods. Our results may help to inform future assay development efforts in overcoming specific challenges with testing and manufacture of products containing live microorganisms.

## Materials and methods

### Organism selection and culture conditions

To facilitate evaluation of the proposed enumeration protocol, a model consortium was developed to simulate the potential diversity of an LBP. Strains from an existing collection of human gut-associated anaerobes (Cheng et al. 2022) were screened for growth on yeast casitone fatty acid agar supplemented with carbohydrates (YCFAC) including 2.0 g/L of glucose, maltose, and cellobiose (Browne et al. 2016; see supplementary methods recipe and preparation). Strain selection was limited to those with existing entries at the species level present in the Bruker BDAL (RUO v. 9) MALDI-TOF MS identification database. Organisms with distinctly recognizable colony morphologies or excessively slow rates of growth were also excluded. A total of eight bacterial strains were selected for use in this study (Table S1) representing four different genera prevalent in human-associated microbial communities including four species of *Bacteroides* (*B. thetaiotaomicron*, *B. finegoldii*, *B. stercoris*, and *B. intestinalis*), two species of *Bifidobacterium* (*Bif. breve* and *Bif. catenulatum*), one *Lactobacillus* (*L. ruminis*), and one *Ruminococcus* (*R. gnavus*). When cultured on YCFAC agar, each of these strains formed medium to small, convex, circular colonies with a white, off-white, or colorless appearance. Colonies were not easily identifiable by visual inspection alone. Axenic glycerol stocks for each selected strain were generated by combining overnight broth cultures 1:1 with sterile YCFAC broth containing 25% glycerol (v/v) for a final glycerol concentration of 12.5%. Glycerol stocks were aliquoted into screw-cap vials and stored at -80 °C for use throughout experimental procedures.

All bacteria were cultured anaerobically in vinyl chambers (Coy Lab Products, Grass Lake, MI, USA) filled with nitrogen gas plus up to 5% hydrogen. Culture incubations were performed at 37 °C without shaking. Culture media and all other materials (e.g., dilution buffers) were placed in the anaerobic chamber at least 18 h before use to reduce to anaerobic conditions. Reduction of media was also confirmed by a color change from light purple/pink to colorless caused by the redox indicator dye resazurin, present in YCFAC media (1 mg/L).

### Preparation of model consortia

Working from frozen glycerol stocks, strains were streaked for isolation onto pre-reduced YCFAC agar and incubated at 37 °C for 24 to 48 h. Isolated colonies were inoculated into 5 mL of pre-reduced YCFAC broth in sterile, round-bottom culture tubes and incubated overnight (~ 18 to 24 h) at 37 °C without shaking. Overnight, broth cultures were homogenized by gentle vortexing, diluted with sterile media to normalize bacterial concentrations, and combined in equal parts (0.5 mL of each diluted culture) to generate model consortia. Initially, cultures were diluted to a common target OD_600_ of 0.10 (± 0.02) before combining up to four different strains. For more complex eight strain mixtures, target OD values were adjusted to obtain a more even distribution of strain-specific concentrations (Table S2).

In total, two batches of two different four-strain consortia and four batches of an eight-strain consortium were generated from independent cultures for experimental enumerations. Additionally, to allow for replicate measurements in a study of assay precision, a single batch of eight-strain mixture was made in the same manner, mixed 1:1 with YCFAC broth containing 25% glycerol (v/v) for a final concentration 12.5% glycerol, aliquoted into sterile screw-cap vials, and stored at -80 °C until use.

### CFU enumeration protocol and calculation

Mixtures and axenic broth cultures were serially diluted tenfold in sterile, pre-reduced PBS (1X PBS pH 7.2, Gibco ThermoFischer). Serial dilutions (10^–3^ to 10^–6^) were used to inoculate 50 μL spread plates on 90 mm YCFAC agar plates. Inoculations were spread using plastic, disposable L-shaped spreaders and an Ohaus inoculation turntable. Spread plates were incubated at 37 °C for two to three days before colony counting and MALDI-TOF MS identification. After determining total CFU values from plates in the dilution series with countable colonies (25 to 250 colonies per plate), isolated colonies from mixture plates were picked at random for identification via MALDI-TOF MS, starting with less crowded dilutions in a series and working up to more crowded plates as needed. To prevent cross-contamination, overlapping or touching colonies were not selected for testing. To reduce the potential for introducing colony selection biases, when possible, all isolated colonies on a given plate were sampled for analysis; otherwise, a random point on the edge of the plate was marked with a pen and colonies were picked outwardly from this point.

The individual CFU values for strains in mixtures (observed CFU) were calculated by normalizing the relative abundance of each strain to the total CFU value according to the following equation:

$$\mathrm{Strain}\;\mathrm{CFU}/\mathrm{mL}\;\left(\mathrm{in}\;\mathrm{mixture}\right)=\frac{\mathrm{Strain}\;\mathrm{Colony}\;\mathrm{ID}\;\mathrm{counts}}{\mathrm{Total}\;\mathrm{Colony}\;\mathrm{ID}\;\mathrm{counts}}\times\mathrm{Total}\;\mathrm{CFU}/\mathrm{mL}$$

The number of colonies picked (i.e., the sampling depth) varied between 115 and 442 colonies per run, depending on the experiment (Table S3). To ensure the purity of overnight cultures and establish a reference measurement for comparison purposes (expected CFU), the OD-adjusted overnight strain cultures were enumerated simultaneously with the mixtures, and CFU values were adjusted according to the dilution factor of each strain in its respective mixture. At least two colonies from each set of reference plates were also selected for MALDI-TOF MS analysis to confirm species-level identities.

### Colony identification via MALDI-TOF MS

A manufacturer-supplied protocol (extended direct transfer method) was used to prepare sampled colonies for MALDI-TOF MS analysis. Briefly, fresh colony material was transferred onto an MBT Biotarget 96 (Bruker Daltonics) target site using a sterile wooden toothpick, overlaid with 1 μL of 70% formic acid (Sigma Aldrich) in HPLC grade water (Sigma Aldrich), and allowed to air dry at room temperature. Once dry, 1 μL of α-Cyano-4-hydroxycinnamic acid (HCCA) matrix solution (10 mg/ml in Bruker Standard Solvent, Sigma Aldrich) was applied and allowed to dry once more before loading sample targets into the MALDI-TOF MS instrument for analysis. The Bruker bacterial test standard (Bruker Daltonics) was included on each Biotarget 96 chip for automated instrument calibration and quality control, per manufacturer recommendations.

Sample mass spectra were acquired on a Bruker MALDI Biotyper (MBT) Smart MALDI-TOF MS instrument (Bruker Daltonics, Billerica, MA, USA) set to detect a molecular mass range of 2 to 20 kDa with a laser frequency of 200 Hz. Bruker flexControl (v 3.4) and MBT Compass (v 4.1) software programs were used for automated instrument control and data acquisition, respectively. Species-level sample identities were determined by the MTB software searching against the Bruker BDAL MSP library database (RUO v. 9). Samples with Biotyper ID scores ≥ 1.70 were accepted for identification, while ID scores < 1.70 were considered unreliable and those samples were excluded from analyses. This cutoff score has been used previously, in conjunction with the formic acid overlay method, for identification of anaerobic bacterial isolates (Hsu and Burnham 2014).

### Data analysis

Expected and observed CFU values were calculated manually in Excel (v. 2108) spreadsheet software and log_10_ transformed prior to analysis. Statistical analyses and data visualization were performed in GraphPad Prism statistical software (v. 9.4.1). Differences of paired log_10_ CFUs were tested parametrically using a paired t-test, with normality of the differences confirmed using the Anderson–Darling normality test. Correlation and unweighted linear regression (least square) analyses were conducted to examine the linear relationship between observed and expected log_10_ CFU values, using an extra sum-of-squares *F*-test to test for significant deviation of slope from the null expectation. Further, agreement analysis was performed using Bland–Altman analysis to calculate the bias and limits of agreement between observed and expected values. A significance threshold of 0.05 was used for all significance testing, and all reported *p*-values are 2-tailed unless otherwise noted in the text. One replicate data pair of *B. intestinalis* was removed from the eight-strain mixture analysis due to a technical error leading to loss of the expected CFU value for this data pair. This point was removed from the graphs and all further statistical analysis.

## Results

### Preliminary testing of four-strain consortia

**Fig. 1:**
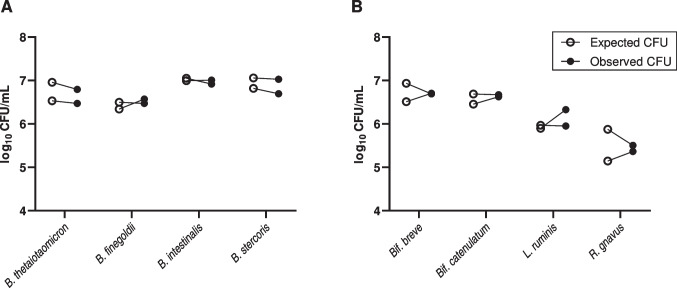
Results from preliminary enumeration of four-strain model consortia. Paired data points (connected) represent expected (open circles) and observed (closed circles) log_10_ CFU values obtained from a single batch enumeration measurement. Consortia consisted of either (A) four different Gram-negative strains from the genus *Bacteroides*, or (B) four different Gram-positive strains from three different bacterial genera. The genus *Bifidobacterium* was abbreviated to “*Bif.*” to avoid confusion with strains from the genus *Bacteroides*

To gage the ability of this protocol to discriminate between closely related bacteria, a mixture containing four different *Bacteroides* species (*B. thetaiotaomicron*, *B. finegoldii, B. intestinalis*, and *B. stercoris*) in approximately equal amounts was enumerated in duplicate. Strong concordance was observed when comparing expected CFU values (axenic enumeration) with the observed CFU values from the mixture (Fig. 1A). Based on enumerations of the individual strain cultures, the expected log_10_ CFU values ranged from 6.34 to 7.06, with an average value of 6.78 ± 0.284 (SD), while observed log_10_ CFU values ranged from 6.47 to 7.03, with an average of 6.75 ± 0.204 (SD). The average difference between paired log_10_ CFU values was 0.0376 ± 0.124 (SD) and not significantly different from zero (*p* = 0.420, paired *t*-test).

To determine whether broad differences in surface structure of bacterial strains (i.e., Gram reaction) would impact enumeration fidelity, a mixture of four Gram-positive bacteria (*Bif. breve*, *Bif. catenulatum*, *L. ruminis*, *and R. gnavus*) was evaluated in a similar fashion to the Bacteroides mixture. The expected log_10_ CFU values for this mixture had a wider range from 5.15 to 6.93, with an average value of 6.19 ± 0.573 (SD), while the observed log_10_ CFU values similarly ranged from 5.36 to 6.71 with an average of 6.23 ± 0.588 (SD). The average difference between observed and expected log_10_ CFU values (Fig. 1B) was 0.045 ± 0.262 (SD) and was not statistically significant (*p* = 0.639, paired *t*-test). Despite the wider range of CFU concentrations in this mixture, concordance with expected CFU values was similar to that of the first experimental mixture. Notably, colony sampling effort was increased to account for the greater range of concentrations (Table S3).

### Testing eight-strain consortium and agreement analysis

**Fig. 2:**
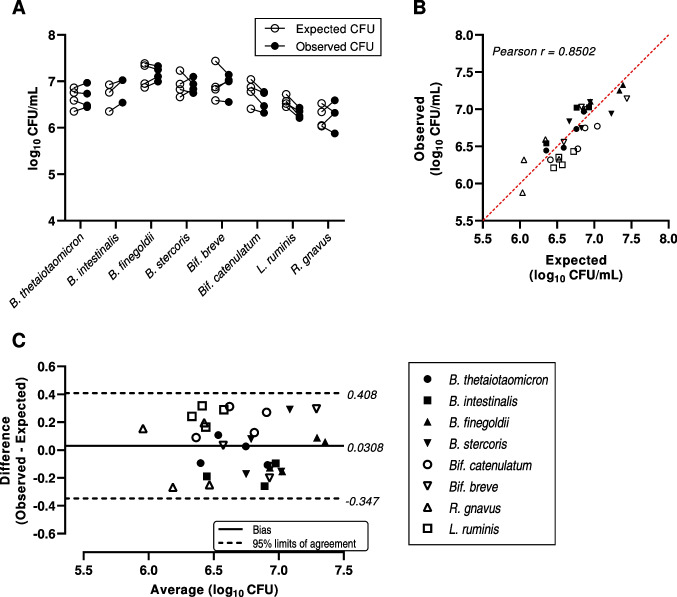
Results and agreement analysis from eight-strain consortium enumeration experiments. (A) Expected and observed log_10_ CFU/mL values with connecting lines indicating paired measurements from the same experimental batch. (B) Correlation analysis of paired expected and observed values with line of identity shown (dashed, red). (C) Bland–Altman plot for analysis of quantitative method agreements with estimates of the estimated bias (solid black line) and 95% limits of agreements (dashed black lines) displayed

For the primary evaluation, an eight-strain consortium was constructed from the component strains of the previous consortia. This mixture consisted of both Gram-positive and Gram-negative organisms representing three distinct bacterial phyla. A total of four batch replicates were tested for assessment of agreement between expected and observed enumeration values for the eight constituent strains. Expected log_10_ CFU values ranged from 6.03 to 7.44 with an overall average of 6.74 ± 0.346, while observed log_10_ CFU values ranged from 5.88 to 7.33, with an average value of 6.71 ± 0.357. The average difference between observed and expected log_10_ CFU values was 0.0306 ± 0.193, and this difference was not statistically significant (*p* = 0.384, paired *t*-test). Observed and expected values were strongly correlated (Pearson *r* = 0.850), and linear a regression analysis found the slope of best fit was not significantly different from 1 (*p* = 0.228, sum-of-squares *F*-test; Fig. 2B).

A Bland–Altman analysis was performed to describe overall agreement between observed and expected log_10_ CFU values for this eight-strain mixture, plotting the differences (observed minus expected) of paired values against their averages (Fig. 2C). The estimated method bias (i.e., the mean of the paired differences between observed and expected values) was 0.0306, with 95% limits of agreement ranging from -0.347 to 0.408. The distribution of paired differences was consistent over the range of average CFU values, and no evidence of proportional bias in the log-transformed data, as the paired differences were normally distributed about the mean throughout the range of average values.

### Measurement precision study

**Fig. 3:**
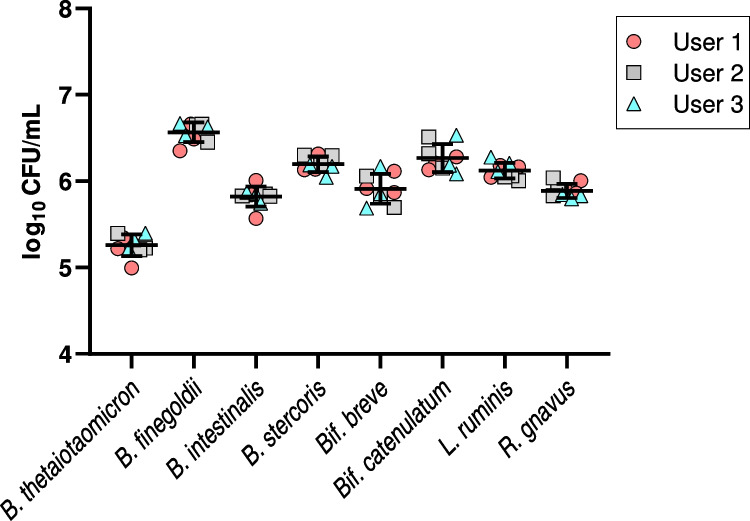
Component strain CFU results from within-laboratory precision study conducted by three different lab users evaluating a single batch of frozen, eight-strain mixture in replicate. Pooled mean and standard deviations shown, with different user values indicated by shape/color of symbols. Overall, differences between users did not contribute significantly to variation in the data (*p* = 0.775, 2-way ANOVA)

To evaluate the repeatability of our experimental measurements, a single batch of eight-strain mixture was made in YCFAC media with 12.5% glycerol (final concentration) and divided into aliquots which were stored at -80 °C. For testing, frozen aliquots were thawed to room temperature under anaerobic conditions before being serially diluted and plated for enumeration and colony identification following the previous mixture enumeration protocol. Three different lab users performed the enumeration protocol three times, with each user replicate performed on a different day, for a total of nine replicate measurements (Fig. 3). All users were trained to run the MALDI-TOF MS for colony identification but had varied levels of experience with the instrument ranging from infrequent or sporadic use to frequent, almost daily use. All eight component strains were recovered and enumerated in each of nine replicate enumeration runs. Average log_10_ CFU values for the eight stains ranged from 5.26 to 6.57, and pooled standard deviation values ranging from 0.067 to 0.193 (Table 1). There was no significant variation between users (*p* = 0.775, 2-way ANOVA).

**Table 1:** Average (SD) of log_10_ strain CFU values obtained from repeated measurements of the same batch of an eight-strain mixture to estimate assay precision (*N* = 3 per user)

	Average log_10_ CFU/mL (SD)			
Strain	User 1	User 2	User 3	Pooled
*B. thetaiotaomicron*	5.19 (0.179)	5.27 (0.104)	5.31 (0.091)	5.26 (0.131)
*B. finegoldii*	6.50 (0.156)	6.59 (0.117)	6.61 (0.072)	6.57 (0.120)
*B. intestinalis*	5.81 (0.223)	5.84 (0.015)	5.82 (0.068)	5.82 (0.135)
*B. stercoris*	6.20 (0.105)	6.26 (0.067)	6.14 (0.078)	6.20 (0.085)
*Bif. breve*	5.97 (0.130)	5.86 (0.183)	5.91 (0.247)	5.91 (0.193)
*Bif. catenulatum*	6.21 (0.076)	6.33 (0.181)	6.27 (0.235)	6.27 (0.177)
*L. ruminis*	6.13 (0.074)	6.04 (0.030)	6.20 (0.084)	6.12 (0.067)
*R. gnavus*	5.92 (0.078)	5.92 (0.106)	5.83 (0.026)	5.89 (0.077)

## Discussion

The motivation for this study was to develop and test a novel method for direct enumeration of multiple bacterial strains from a defined consortium simultaneously. This protocol uses MALDI-TOF MS-based rapid colony identification to determine the proportional abundances of each constituent strain and multiplying these values by the total CFU present in the sample to determine absolute CFU values. In addition to finding strong positive correlations between the experimental and reference methods (Fig. 2B), no evidence of systematic or proportional biases was found in the Bland–Altman agreement analysis. We further demonstrated the repeatability and consistency of this enumeration method across nine replicate measurements of a single batch of eight-strain mixture, finding only modest differences in standard deviation values for the eight component strains.

Advantages of the approach developed in this study include the relatively low cost per sample and rapid turn-around times of MALDI-TOF MS colony identification methods (Dhiman et al. 2011; Singhal et al. 2015). In our lab, manual preparation of 96-sample MS target chips, including colony picking, deposition, and drying time of formic acid and matrix solutions, took ~ 40 to 60 min, depending on the user’s level of experience. This approach also quantifies viable organisms in terms of CFU, which remains the most widely recognized measure of live microbials (US Food and Drug Administration 2016, US Food and Drug Administration 2018). Potential barriers to implementation could include high start-up and maintenance costs of MALDI-TOF MS instrumentation as well as limitations in available reference libraries (Haider et al. 2023). While multiple studies have reported overall cost savings of MALDI-TOF MS use in routine clinical testing over traditional microbiological methods (Gaillot et al. 2011; Tran et al. 2015), cost effectiveness would likely depend on the rate of use and unit costs of alternative identification methods for the target organisms. While this study utilized bacterial strains with species-level MSPs already present in the Bruker BDAL MSP library, specific reference spectra can be generated for novel strains or species of interest and grouped into customized libraries (Hou et al. 2019). MALDI-TOF MS characterization might also serve as part of the comprehensive strain identity and characterization requirements for microorganisms used as active substances in LBPs (Paquet et al. 2021).

MALDI-TOF MS methods are also potentially amenable to automation through use of robotic colony picking and sample preparation equipment. Automation of sample preparation can reduce the amounts of consumable materials and hands-on time required for sample preparation and may also improve consistency and repeatability of colony identification results (Chudejova et al. 2017; Heestermans et al. 2022). In a previous validation study examining over 500 clinical isolates, Chudejova et al. (2017) found automated sample preparation using the MALDI Colonyst robot improved overall identification scores and reduced error rates among both bacterial and fungal isolates when compared with manual sample deposition methods. While our study utilized only one MALDI-TOF MS instrument for MS-based colony identification, other instruments may perform at comparable levels (Pence et al. 2014; Levesque et al. 2015; Porte et al. 2017; Brown-Elliott et al. 2019; Park et al. 2021).

One limitation of this study is that we chose not to conduct statistical power analyses for determining appropriate sampling depths prior to experimental testing, nor was the sampling depth strictly controlled between experimental replicates (Table S3). Except for the precision study experiments, replicate plates from mixed enumeration studies were sampled to completion rather than to a pre-specified number of colonies. Various considerations would factor into calculation of the appropriate sample sizes needed for an assay of this format, such as the intended purpose of the test (e.g., product release, process control, and stability testing), the specific composition of a microbial consortium or LBP, and the precision and/or accuracy required under the applicable regulatory framework (US Food and Drug Administration 2016, Dreher-Lesnick et al. 2017). Meaningful power or sample size calculations for this method would need to account for multiple potential sources of sampling error related to the reported CFU values and composition of the product being tested.

Despite limitations in scope and scale, this study demonstrates the feasibility of a newly developed method to simultaneously generate accurate and repeatable CFU values for up to eight bacterial strains from a defined consortium. Accurate identification and quantification of living microbial components in food and/or drug products are essential parts of product characterization for dosing optimization, clinical trial design, and general assessments of product safety, potency, and stability. As a proof-of-concept, this study should help inform future efforts to develop accurate and repeatable CFU enumeration protocols related to LBPs, probiotic supplements, and food products containing multiple live microbial strains.

## Supplementary Information

Below is the link to the electronic supplementary material.

Supplementary file1 (PDF 261 KB)

## Data Availability

Data generated and/or analyzed as part of this study are available upon reasonable request from the corresponding author.
