# Supplementary material for: Application of MALDI-TOF MS for enumerating bacterial constituents of defined consortia
Source: Appl Microbiol Biotechnol. 2023 May 6;107(12):4069–77. doi: 10.1007/s00253-023-12558-5 (PMC10238304; doi:10.1007/s00253-023-12558-5)
Supplement: Supplementary file 1 — Supplementary file1 (PDF 261 KB) [file 253_2023_12558_MOESM1_ESM.pdf]

Supplementary Information

Applied Microbiology and Biotechnology

Manuscript Title:

Application of MALDI-TOF MS for enumerating bacterial constituents of defined consortia

Author Information:

Michael P. Coryell*[a], Rosa L. Sava*[a], Jessica L. Hastie[a], **Paul E. Carlson Jr.[a] (Corresponding Author)**

*These co-authors contributed equally to the manuscript

Affiliations
[a] Division of Bacterial, Parasitic and Allergenic Products; Office of Vaccines Research and Review, Center for Biologics Evaluation and Research, US Food and Drug Administration.
Silver Spring, Maryland, USA.

Corresponding author:
Paul E. Carlson, Jr.
Email: paul.carlson@fda.hhs.gov

Supplementary Materials and Methods

Preparation of YCFAC culture medium.

The recipe for yeast casitone fatty acid medium supplemented with carbohydrates (YCFAC) culture media used in this study was adapted from Browne et al. (2016), where the supplementation of YCFA base medium (Duncan et al. 2002) with carbohydrates, including glucose, maltose, and cellobiose, was used to encourage growth of previously uncultivated organisms from the human fecal microbiome. The complete YCFAC culture media used in this study contained the following ingredients: casitone (10 g/L), yeast extract (2.5 g/L), glucose (2.0 g/L), cellobiose (2.0 g/L), maltose (2.0 g/L), potassium phosphate monobasic (0.45 g/L), potassium phosphate dibasic (0.45 g/L), sodium chloride (0.90 g/L), ammonium sulfate (0.90 g/L), magnesium sulfate heptahydrate (90 mg/L), calcium chloride dihydrate (90 mg/L), glacial acetic acid (33 mM), propionic acid (9.0 mM), isovaleric acid (1.0 mM), isobutyric acid (1.0 mM), hemin (10 mg/L), resazurin (1.0 mg/L), L- cysteine HCl (1 g/L), ATCC MD-VS vitamin supplement (10 ml/L), and agar (15 g/L, omitted from broth formulation).

Prior to making media, stock solutions of hemin (10 mg/mL in 1N NaOH), resazurin (1.0 mg/mL in 1x PBS, filter sterilized), L-Cysteine HCl, anhydrous (0.5 g/mL in water, filter sterilized) were prepared and stored protected from light at 4 °C, 4 °C, and -20 °C respectively. The vitamin supplement solution came pre-sterilized in 10 mL aliquots from ATCC and was stored at -20 °C. For media preparation, all reagents except for cysteine and vitamin supplement solutions were dissolved in DI water with stirring and heat, starting with dry reagents before adding volatile fatty acids, hemin, and resazurin. After cooling to room temperature, pH was adjusted to a range of 6.7 to 6.8 with NaOH (10 N stock solution in water) and gentle stirring. Media were sterilized in an autoclave at 121 °C for 30 minutes while cysteine and vitamin supplement solutions were thawed to room temperature and mixed on a benchtop vortex. In the event of precipitate formation, cysteine aliquots were warmed to 37 °C and thoroughly vortexed to ensure complete re-dissolution. Use aseptic technique, cysteine and vitamin supplement solutions were added to

cooled media (<60 °C), while stirring gently. After 2-5 minutes of continuous mixing, agar medium was poured by hand into 90 mm sterile plastic Petri dishes (~25 to 30 ml per plate). Prior to use, plates and broth containers were placed into the anaerobic chamber and allowed to reduce overnight (18 to 24 hour). Agar depth was not mechanically standardized, but plates were visually inspected for consistency of depth.

Supplementary Tables

Table S1 List and descriptions of all bacterial strains used in this study.

Organism	Strain designation	Collection/No.	Phylum	Gram reaction
Bacteroides thetaiotaomicron	VPI-5482	ATCC 29148	*Bacteroidetes*	Negative
Bacteroides finegoldii	119	DSM 17565	*Bacteroidetes*	Negative
Bacteroides stercoris	VPI B5-21	ATCC 43183	*Bacteroidetes*	Negative
Bacteroides intestinalis	341	DSM 17393	*Bacteroidetes*	Negative
Bifidobacterium breve	S1	DSM 20213	*Actinobacteria*	Positive
Bifidobacterium catenulatum	B669	DSM 16992	*Actinobacteria*	Positive
Lactobacillus ruminis	E 194e	ATCC 25644	*Firmicutes*	Positive
Ruminococcus gnavus	VPI C7-9	ATCC 29149	*Firmicutes*	Positive

Table S2 Adjusted OD_{600} values used when preparing eight-strain consortium from overnight cultures. Target values chosen using standard curve method to reach ~7.3 Log_{10} CFU per mL.

Study strain	Target OD600
B. thetaiotaomicron	0.07
B. intestinalis	0.13
B. finegoldii	0.14
B. stercoris	0.06
Bif. breve	0.07
Bif. catenulatum	0.08
L. ruminis	0.33
R. gnavus	0.61

Table S3 Experimental sampling depths in terms of the numbers of colonies screened and identified per run, broken down by experimental data figure and replicate. An ID score of ≥ 1.7 was used as the cutoff for positive identification in this study, with colony identification failed when returning an ID score <1.70, or when no peaks were identified due to insufficient sample material on the target.

Data figure	No. strains	Experimental replicate	Colonies screened	Colonies identified (ID score ≥ 1.70)	Identification rate
1a	4	1	115	112	97.4%
1a	4	2	118	118	100.0%
1b	4	1	180	162	90.0%
1b	4	2	211	210	99.5%
2	8	1	254	241	94.9%
2	8	2	316	279	88.3%
2	8	3	442	399	90.3%
2	8	4	251	245	97.6%
3	8	1.1	368	360	97.8%
3	8	1.2	368	363	98.6%
3	8	1.3	368	340	92.4%
3	8	2.1	368	367	99.7%
3	8	2.2	368	364	98.9%
3	8	2.3	368	355	96.5%
3	8	3.1	368	318	86.4%
3	8	3.2	368	297	80.7%
3	8	3.3	368	318	86.4%
**Totals**	**test**	**test**	**5199**	**4848**	**93.2%**

References

Browne HP, Forster SC, Anonye BO, Kumar N, Neville BA, Stares MD, Goulding D, Lawley TD (2016) Culturing of 'unculturable' human microbiota reveals novel taxa and extensive sporulation. Nature 533(7604): 543-546. https://doi.org:10.1038/nature17645

Duncan SH, Hold GL, Harmsen HJM, Stewart CS, Flint HJ (2002) Growth requirements and fermentation products of Fusobacterium prausnitzii, and a proposal to reclassify it as Faecalibacterium prausnitzii gen. nov., comb. nov. Int J Syst Evol Microbiol 52(Pt 6): 2141-2146. https://doi.org:10.1099/00207713-52-6-2141
